# Measuring the building blocks of everyday cognition: executive functions and relational reasoning

**DOI:** 10.3389/fpsyg.2023.1219414

**Published:** 2023-09-27

**Authors:** Lindsey Engle Richland, Hongyang Zhao

**Affiliations:** School of Education, University of California, Irvine, Irvine, CA, United States

**Keywords:** executive function, relational reasoning, cultural context, problem solving, WEIRD samples

## Abstract

Measurement of the building blocks of everyday thought must capture the range of different ways that humans may train, develop, and use their cognitive resources in real world tasks. Executive function as a construct has been enthusiastically adopted by cognitive and education sciences due to its theorized role as an underpinning of, and constraint on, humans’ accomplishment of complex cognitively demanding tasks in the world, such as identifying problems, reasoning about and executing multi-step solutions while inhibiting prepotent responses or competing desires. As EF measures have been continually refined for increased precision; however, they have also become increasingly dissociated from those everyday accomplishments. We posit three implications of this insight: (1) extant measures of EFs that reduce context actually add an implicit requirement that children reason using abstract rules that are not accomplishing a function in the world, meaning that EF scores may in part reflect experience with formal schooling and Western, Educated, Industrialized, Rich, Democratic (WEIRD) socialization norms, limiting their ability to predict success in everyday life across contexts, (2) measurement of relational attention and relational reasoning have not received adequate consideration in this context but are highly aligned with the key aims for measuring EFs, and may be more aligned with humans’ everyday cognitive practices, but (3) relational attention and reasoning should be considered alongside rather than as an additional EF as has been suggested, for measurement clarity.

## 1. Introduction

Executive function (EF) is a construct that has taken on great attention in cognitive science as well as in educational and psychological literatures aiming to train and improve children’s developmental trajectories, due to its theorized centrality to human cognition as a building block, and accordingly as a capacity limiter, in higher cognitive function. As such, EFs are theorized to predict individual differences in human reasoning, problem solving, and learning, and there is much data to support this inference, though the specific relationships between individual EFs and these key processes are somewhat variable (Bull and Scerif, 2001; Bull and Lee, 2014; Simms et al., 2018). Indeed the literature on EFs is highly variable, and it is clear that task-specific constraints and affordances are impactful on measurements, in part due to task impurity such that most tasks involve multiple types of EF demands (Burgess, 1997; Phillips, 1997), and in part because of potential lack of clarity about the nature of composite EF skills (Doebel, 2020).

At the same time, there are often wide disparities between the ways that EFs are measured, the everyday skills they are intended to explain, and the ways they are used and invoked by educators invested in improving knowledge and skills. This is important theoretically for measurement but also for guiding recommendations for training EFs. When measurements are misalignments to the everyday skills EFs are designed to explain and constrain, training recommendations stem from these measures rather than usage in the world. One consequence is the potential low likelihood that trained gains would thus transfer to everyday practices.

A second, less well considered consequence is that cultural norms and expectations that are embedded in the creation of EF tasks may be particularly misaligned with the human reasoning and problem solving performed by individuals in non-Western, Industrialized, Educated, Rich and Democratic (WEIRD; Henrich et al., 2010), contexts. This may lead to measurement of skills that are not predictive of these individuals’ everyday performance and may suggest training practices that could be inefficient or counter to extant practices that are indeed predictive of success in real world contexts.

This manuscript focuses on elaborating these concerns, and poses approaches to responding to this challenge. In particular we focus on illuminating culturally valanced assumptions that are embedded in many EF tasks, and suggest that relational reasoning and relational attention are cognitive measures that incorporate but do not seek to reduce EFs into their base cognitive units, may in fact be closer to meeting the second two goals highlighted above – explaining everyday cognitive behaviors and limits, and supporting training for regulating one’s behavior to best make use of one’s limited cognitive resources.

## 2. Defining executive functions

EFs are commonly defined as the limited capacity cognitive processing system that deploys resources to perform cognitive tasks and regulate the dynamics of human cognition (see Diamond, 2006; Miyake and Friedman, 2012). Within EF, the dominant model centers on three primary subsystems that include Working Memory (WM), the resources for holding information active within attention and manipulating that information (Engle and Kane, 2004), attentional control, or inhibitory control, the processes of controlling attention away from irrelevant information and inhibiting prepotent responses (Diamond, 2002), and task switching, the ability to regulate attention and execution of task rules when moving between two or more tasks (Miyake et al., 2000; Zelazo et al., 2004).

EFs are theorized to be integral to intelligent behavior (Carpenter et al., 1990; Little et al., 2014), as well as school-based achievement skills (Best et al., 2011) including mathematics (Bull and Lee, 2014) and reading (van der Sluis et al., 2007; Kim, 2020). Importantly to broad everyday impact, EFs indicated to be integral to human higher cognitive functions such as reasoning and problem solving (Morrison et al., 2004; Richland et al., 2006; Krawczyk et al., 2008; Richland and Burchinal, 2013).

At the same time, measurement of EF skills is not straightforward, and complications have arisen because measures that are ostensibly of the same process do not always correlate, and at the same time, many EF tasks involve shared skills, which is difficult to disentangle (Miyake et al., 2000; Snyder et al., 2015).

In the aim to resolve this challenge and produce tasks that have removed the interference of other EFs as well as everyday knowledge and experiences; however, the field has also shaped these tasks in ways that may not reliably reflect all children’s skills.

## 3. EFs across cultural populations

Growing evidence has documented that cultural context and socialization practices profoundly impact cognitive development, but even so, models of key psychological constructs such as EFs continue to be primarily developed and refined on samples of children from WEIRD societies, which represent only a small portion of the world’s population (12%; Henrich et al., 2010). This sampling bias may be particularly consequential in a theoretical domain such as EFs, where socialization practices across communities may have direct implications for children’s opportunities for displaying their ability to enact problem solving, holding information in mind, managing and switching tasks, and inhibiting prepotent responses.

We posit that the tendency for most standardized, field accepted measures of EF to require children to manipulate arbitrary rules to solve non-consequential tasks may have led them to be broadly aligned with many skills taught within WEIRD formal educational and socialization routines, and misaligned with socialization routines identified in other communities. For example, in rural and indigenous Latine communities, children are highly autonomous and are not expected to follow verbally articulated arbitrary rules without a clear rationale or consequence (see Gaskins, 2000; Correa-Chávez and Rogoff, 2009; Ochs and Izquierdo, 2009; Alcalá et al., 2018; Kulis et al., 2019). In another example building on measurement of delayed gratification abilities, Japanese children were found to wait longer than American children for food, but not for gifts. Such different patterns of self-control could be due to cultural differences (Yanaoka et al., 2022). In Japan, mealtime is often considered as a communal and social event. It is customary to wait until everyone is seated and ready before starting a meal. Waiting for everyone to be present before eating is considered polite and demonstrates consideration for others. However, in many communities within American society these values are less associated with mealtime. Instead, many U.S. children may be more used to waiting to open gifts, for instance when everyone is present at a holiday gathering such as Christmas. This practice allows for the family or group to share and celebrate joy and excitement as gifts are opened together. These examples provide evidence that cultural routines and socialization can play an important role in influencing attentional control behaviors and must be considered when conceptualizing and measuring EF. Recognition of this problem is important to the field.

Theoretically, the under-considered role of arbitrary rules in EF tasks and cultural context could have led to models of reasoning and EF that are culturally specific and could explain some lack of shared variance across many EF tasks, as well as the low performance among lower wealth and less educated participants. The literature linking poverty to EFs is robust (Dahlman et al., 2013; Pluck et al., 2018), yet at the same time, Dahlman et al. (2013) find instead that unhoused children in Bolivia scored significantly higher on an EF flexibility and planning tasks than children with more stable homes (Dahlman et al., 2013), so SES may be confounded with participants’ alignment with cultural routines implicit within EF task measurement.

Building strong and effective EF skills in the service of strong problem solving and reasoning has been posited to be one of the most crucial 21st century skills, meaning better understanding how to capitalize on children’s assets to support their development has the potential for powerful and broad impacts on children’s cognitive development. Rather than pushing first/s generation Latine children’s routines away from their everyday practices, for example, it could theoretically instead be important to support and enhance children’s participation and autonomy in daily tasks.

## 4. Relationship between EFs and relational reasoning

While EFs have gained attention due to their role as a building block of higher cognition and as being crucial to the skills and practices defined as central to success in the modern world of technology, innovation, and flexible problem solving, relational reasoning has long been studied as a building block foundation to these same skills (see Gentner and Holyoak, 1997; Markman and Wood, 2009; Richland and Simms, 2015). Relational reasoning is the process of drawing relational correspondences across representations, enabling reasoners broad opportunities including to make inferences from known information to novel problems or contexts, to recognize opportunities to transfer solutions from one problem to another, to build understanding of concepts or abstractions. These are underpinnings of innovation, problem solving, educational learning and expertise, higher order thinking and inferences about everyday phenomena (Markman and Wood, 2009; Richland and Simms, 2015; Zhao et al., 2021).

Starr et al. (2023) have argued that relational reasoning should be considered one of the EFs. They provide a compelling analysis of canonical EF measures and relational reasoning, finding a high correlation between relational reasoning and most of the EF measures, but also that this task better explained variance in math fluency and fraction comparison task performance than the EF measures.

We concur that measuring relational reasoning is crucial to understanding the building blocks of human cognitive activity, with relational reasoning being a core component of expertise in many educational domains (Richland and Simms, 2015; Alexander, 2019; Bunge and Leib, 2020; Zhao et al., 2021), and theorize that relational reasoning measures may be more likely to capture children and adults’ skills at managing attention and information in the world to accomplish tasks than traditional EF tasks. They may be also more likely than many EF measures to generalize across cultural contexts when in problem solving form, thereby being closer to characterizing what makes humans successful in varied contexts including non-WEIRD environments.

**Figure 1:**
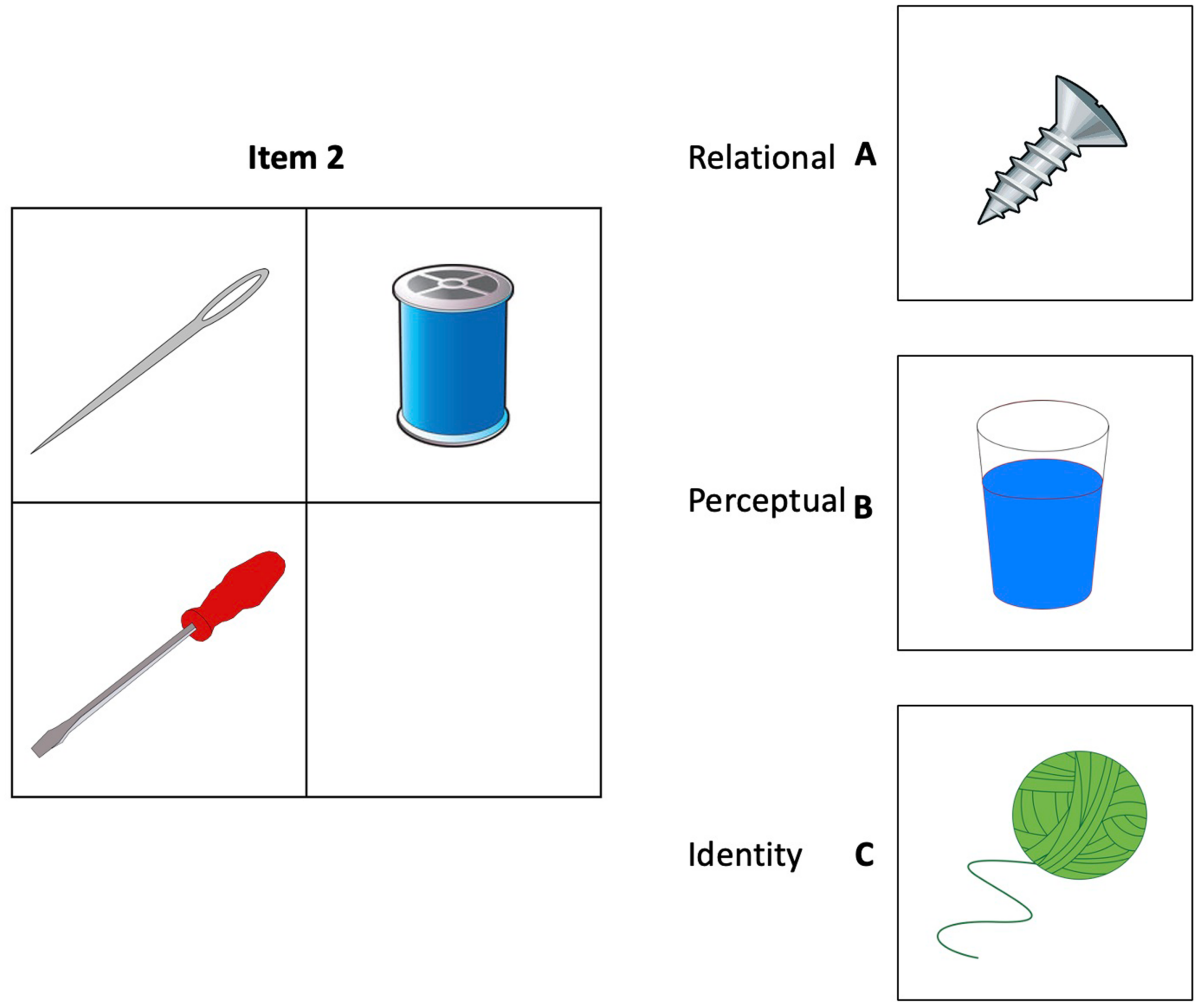
A sample item from relational reasoning measures.

At the same time, we suggest that adding relational reasoning to the characterization of EFs will have the effect of perpetuating and expanding the challenges in developing precision in measurements that should correlate highly across EF tasks. Relational reasoning has by its nature levels of difficulty that may not function as linear, and in that way functions differently than other EFs. One type of difficulty in relational reasoning is the need to focus one’s attention on relational, rather than other types of similarity, including object correspondences, association, or perceptual similarity (see Rattermann and Gentner, 1998; Figure 1), where the D term of a matrix could be filled by relational or perceptual similarity (Simms and Richland, 2019). Relational attention may shift with a reasoner’s expertise in the relevant knowledge-base, which changes the nature of reasoners’ attention to the relational content of a task (Chi et al., 1981). As knowledge increases, reasoners may shift from attention to surface features and object-level correspondences to relational correspondences (see Gentner, 1988; Starr et al., 2018; Thibaut et al., 2022).

Relational attention can also be manipulated by task goals and recent reasoning experience (Holyoak and Thagard, 1997; Vendetti et al., 2014; Walker et al., 2018; Simms and Richland, 2019), and may be in itself an individual difference that predicts learning and task ability (Zhao et al., under review). Relational attention, described by Vendetti et al. (2014) as a relational mindset, refers to the likelihood of noticing relational correspondences versus perceptual or featural similarity (see Vendetti et al., 2014; Simms and Richland, 2019), when there is not a specific cue to direct attention to object or relational correspondences.

Secondly, relational reasoning tasks vary by relational complexity, which again may not function as linear difficulty on an individual basis, but rather change in relation to individual differences in other EF capacities. These seem to function with a baseline, such that with adequate EFs for a task, reasoners’ performance will vary minimally across levels of relational complexity, while with not adequate EFs, reasoners may make relational errors, or may reason in qualitatively different ways, focusing on perceptual similarity rather than relational similarity (see Gentner and Rattermann, 1991; Richland et al., 2006; Krawczyk et al., 2008; Thibaut et al., 2010; Simms et al., 2018).

Thus, the cognitive resources of relational reasoning and EFs are distinct, and relational reasoning should be considered by researchers aiming to investigate the cognitive building blocks underlying individual differences in complex thought and intelligent behavior, but they are not independent and is productive to measure alongside EFs (see also Richland and Morrison, 2010).

There are also variations in the capacities involved in relational reasoning measured by different tasks. Verbal and non-verbal relational reasoning relate differently to verbal skills, and scores measured by relational tasks themselves may vary based on the form of the comparisons themselves (TORR, TORRJr: Zhao et al., 2021).

## 5. Training EFs: building on everyday assets and use of EFs in context

The developmental trajectory of children’s EF skills suggests these grow and shift over the lifespan (see Anderson, 2002; Zelazo et al., 2004), yet the mechanisms driving changes are not well understood, which has implications for policies and protections for encouraging its growth. The vast majority of explicit EF training programs involve repeated experiences with cognitively demanding training programs such as repeated practice on the dual n-back task (see Jaeggi et al., 2020), and many such studies find gains on the same EF task trained, but inconsistent or sometimes no transfer to new formal EF tasks (Doebel, 2020; Niebaum and Munakata, 2023). This suggests that if the ultimate goal of building EF skills is to support youth’s ability to perform tasks such as handling complexity in reasoning, inhibiting misleading pre-potent responses, and switching between taxing everyday tasks, perhaps EF training should take place by engaging in these types of tasks.

Some studies provide evidence that there may be productive gains for EFs as measured in traditional tasks by engaging in everyday activities such as sports or certain types of preschool curricula (Diamond, 2006; Niebaum and Munakata, 2023). Importantly, other seemingly mundane everyday practices that are not extra-curricular (and thus tied to available SES resources) but rather are tied to home work have not been investigated but seem to involve the same types of cognitive resource work, such as remembering long lists of groceries while going to and purchasing at the market, planning multi-step sequences while cooking or fixing equipment. Children’s involvement in these practices varies dramatically across cultural communities (see Alcalá et al., 2021), and thus may be underrecognized but potent means for training EF skills. At the same time, individual differences in children who display strong skills on activities such as shopping as noted above, may not be scored accordingly by a working memory measure requiring children to perform a task while retaining long lists of arbitrary letters, due to factors discussed here that may artificially limit performance, most notably because these children may treat the importance and goals of these tasks differently.

There is some evidence to support the role of cultural practices as unrecognized assets for EF training. Previous research on EF skills suggests that everyday bilingualism can lead to gains in EF skills, particularly on tasks requiring cognitive flexibility and managing conflicting attentional demands (Carlson and Meltzoff, 2008; Bialystok, 2011). However, there might be other cultural factors, in addition to bilingualism, that contribute to their EF skills. For example, Chinese-American immigrant children’s performance on some measures of executive control was predicted only by proficiency in Chinese, suggesting that perhaps higher fluency in Chinese could be related to greater experience with traditional Chinese values of obedience, behavioral control, and self-restraint, which would be causal to developing the higher EF skills (Chen et al., 2014).

### 5.1. Cultural variability in autonomy

A developmental mechanism that has not yet been considered broadly to play a role in the measurement and development of EFs is the known variability in children’s level of autonomy and management of household tasks across cultural communities (see Gaskins, 2000; Alcalá et al., 2014). Management of household tasks often requires decision-making about key goals and tasks that are necessary to accomplish and execute these tasks. Mechanistically, this often involves holding high amounts of information in mind while solving problems, inhibiting prepotent responses to one stimulus in favor of persisting on another task, or fluidly switching between tasks that must be completed. These are processes that seem to require both relational reasoning/ problem solving and high levels of EFs, and thus may be a potent training regime that has not yet been considered as such.

Cross-cultural research has documented a wide range in ideologies about the level of autonomy and control that parents expect children to maintain, visible as the level of work and initiative that children contribute to household work and other community activities (Gaskins, 1996, 2000; Gaskins and Paradise, 2010; Alcalá et al., 2014). For example, a study of first and second generation Latine children in California found higher levels of help at home on their own initiative than observed in European American families, while keeping in mind the needs of the group and help when needed (Alcalá et al., 2018). A study of indigenous children revealed more time spent time in household work and engaging in free play, setting their own agenda, while European-American children had less access to work and were more likely to participate in activities organized and managed by adults (Ochs and Izquierdo, 2009; Cervera, 2016). In a recent study on the impact of COVID on child development across cultures found clear cultural differences in how families organized children’s level of autonomy and participation in the household (Alcalá et al., 2021).

Additionally, having the experience of making consequential decisions and solving real problems raises children’s expectation that they can and should make real decisions about when and whether to engage one’s reasoning and EF resources in any given task. The implication for standard psychometric measures of EF is that these children may be less likely to do so when the task rules are arbitrary, and any actual gain is not recognizable. In homes where children’s lives are organized and guided by adults, children may become highly skilled at following instructions, while in homes where children take initiative and manage tasks, children may become highly skilled at making their own decisions about how to manage complex tasks, determining goal directed behavior and holding constraints in mind while acting to perform other tasks. These different modes of engaging with the world may differently affect performance on psychometric tasks regardless of EF capacities (Barker et al., 2014).

## 6. Conclusion

We face a pressing need to understand the building blocks of everyday thinking and learning, to better know how to prepare youth to succeed in a complex and changing world (National Academies of Sciences, Engineering, and Medicine, 2018). Children learn as they engage in culturally meaningful activities (Rogoff, 2014), supported by a set of dynamic processes that need to be coordinated for learning to occur, including attention, emotional regulation, and inhibition of incorrect or inadequate responses. Measurement of the building blocks of everyday cognition must capture the range of different ways that humans may build and use attentional control in real world tasks, and relational attention and reasoning is an underdeveloped field for measuring these individual differences. EF measurements must also be better aligned with the range of cultural practices humans use them for, with one key aspect being to recognize the cultural constraints present in the use of tasks that require manipulating arbitrary rules – a hallmark of contemporary EF measurement.

## Data availability statement

The original contributions presented in the study are included in the article/supplementary material, further inquiries can be directed to the corresponding author.

## Author contributions

LR took lead on formulating the manuscript focus and text. HZ contributed to all study ideas and manuscript writing. All authors contributed to the article and approved the submitted version.
